# Quantitative futility in emergency laparotomy: an exploration of early-postoperative death in the National Emergency Laparotomy Audit

**DOI:** 10.1007/s10151-022-02747-1

**Published:** 2023-01-07

**Authors:** H. Javanmard-Emamghissi, B. Doleman, J. N. Lund, J. Frisby, S. Lockwood, S. Hare, S. Moug, G. Tierney

**Affiliations:** 1 Department of Medicine and Health Science, University of Nottingham at Derby, Royal Derby Hospital, Derby, UK; 2 Department of Palliative Care Medicine, Royal Derby Hospital, Derby, UK; 3 Department of Colorectal Surgery, Bradford Royal Infirmary, Bradford, UK; 4 Department of Anaesthesia, Medway Maritime Hospital, Kent, UK; 5 Department of Colorectal Surgery, Royal Alexandra Hospital, Paisley, UK; 6 Department of Colorectal Surgery, Royal Derby Hospital, Derby, UK

**Keywords:** Emergency laparotomy, Emergency general surgery, Futility, Mortality, Informed consent, Shared decision-making, Frailty

## Abstract

**Background:**

Quantitative futility is an appraisal of the risk of failure of a treatment. For those who do not survive, a laparotomy has provided negligible therapeutic benefit and may represent a missed opportunity for palliation. The aim of this study was to define a timeframe for quantitative futility in emergency laparotomy and investigate predictors of futility using the National Emergency Laparotomy Audit (NELA) database.

**Methods:**

A two-stage methodology was used; stage one defined a timeframe for futility using an online survey and steering group discussion; stage two applied this definition to patients enrolled in NELA December 2013–December 2020 for analysis. Futility was defined as all-cause mortality within 3 days of emergency laparotomy. Baseline characteristics of this group were compared to all others. Multilevel logistic regression was carried out with potentially clinically important predictors defined a priori.

**Results:**

Quantitative futility occurred in 4% of patients (7442/180,987). Median age was 74 years (range 65–81 years). Median NELA risk score was 32.4% vs. 3.8% in the surviving cohort (*p* < 0.001). Early mortality patients more frequently presented with sepsis (*p* < 0.001). Significant predictors of futility included age, arterial lactate and cardiorespiratory co-morbidity. Frailty was associated with a 38% increased risk of early mortality (95% CI 1.22–1.55). Surgery for intestinal ischaemia was associated with a two times greater chance of futile surgery (OR 2.67; 95% CI 2.50–2.85).

**Conclusions:**

Quantitative futility after emergency laparotomy is associated with quantifiable risk factors available to decision-makers preoperatively. These findings should be incorporated qualitatively by the multidisciplinary team into shared decision-making discussions with extremely high-risk patients.

**Supplementary Information:**

The online version contains supplementary material available at 10.1007/s10151-022-02747-1.

## Introduction

Futility occurs when ‘there is a goal, there is an action and activity aimed at achieving this goal and there is virtual certainty that the action will fail in achieving this goal’ [1]. Quantitative futility is the scientific assessment of the probability of the failure of a treatment [2], demonstrated by evidence-based risk prediction models like the National Surgical Quality Improvement Program (NSQIP), National Emergency Laparotomy Audit (NELA) and Portsmouth Physiological Operative Severity Score (P-POSSUM) [3–5]. These risk prediction models calculate the percentage risk of 30-day mortality after emergency laparotomy in order to guide clinical decision-making [3–5]. The concept of futility in surgery is highly controversial [6], but it is incontrovertible that in surgery there is an important balance to maintain between under and over treatment. Treatment that is not in concordance with a patient’s goals and priorities has been associated with post-traumatic stress disorder and complex grief for their families, and is associated with moral distress and burnout in clinicians [7–10].

For those who do not survive, an emergency laparotomy has provided negligible therapeutic benefit. This may represent a missed opportunity in the multidisciplinary discussion on goals of treatment to consider changing the focus of active management to palliation. Significant quality improvement work, led by NELA, has improved the 30-day mortality rates of emergency laparotomy patients in England and Wales [11, 12]. In contrast, very little work has explored early-postoperative mortality, arguably a group for which emergency laparotomy has provided the least benefit [12, 14, 15]. Work that has attempted to define quantitative futile surgery and examine the patient group that has futile surgery, has used different definitions of futility and has failed to incorporate frailty, which has become a key marker for poor postoperative outcomes [12, 14, 15].

The aims of this study were to define a timeframe for quantitative surgical futility in the emergency laparotomy setting and to apply that definition to the NELA dataset, allowing characterisation of this patient population and exploration of predictors of early mortality.

## Materials and methods

We used a two-stage methodology. Stage 1 aimed to seek a consensus definition of a timeframe for futile emergency surgery using an online survey and a scoping review followed by a steering group consensus discussion. Stage 2 applied this accepted timeframe to the NELA database for analysis.

### Stage 1

Key stakeholders were invited through convenience sampling to complete an online cross-sectional survey through a link distributed to members by the Association of Surgeons of Great Britain and Ireland, Age Anaesthesia and promoted on social media. The survey was entirely anonymous and open to all clinicians with any experience in the care of emergency laparotomy patients, including allied health professionals. The survey was hosted on Google Forms (Google LLC, CA, USA), and was open for responses for a 4-week period in September 2020. After low engagement from geriatricians, further promotion was carried out through the British Geriatrics Society and the survey was re-opened for responses for a further 4 weeks in February 2021. The online software prevented each participant from being surveyed more than once. A response rate was not calculable due to the method of advertising through social media.

Clinicians were asked to rate their responses to statements about surgical futility on a five-point Likert scale and to rank factors contributing to this decision. Seven questions were trialled by a panel of trainee and consultant anaesthetists and surgeons, for readability, non-ambiguity and content and are listed in Appendix. For the scoping review, a search of the published literature on surgical futility was performed and is fully described in a separate publication [16].

The timeframe of surgical futility was then determined by the majority result from the survey and the scoping review being debated within the steering group. The steering group consisted of a trainee surgeon and anaesthetist, four consultant surgeons, a palliative medicine consultant and anaesthetic consultant. This definition was then taken forward into Stage 2.

The survey was reported according to the CROSS reporting guidelines [17].

### Stage 2

The NELA database was analysed using the definition of futile surgery. NELA is a mandatory national audit in England and Wales designed to describe processes of care for, and improve the quality of care provided to patients undergoing emergency laparotomy [11]. Data on patient presentation and outcomes such as mortality and length of stay are captured locally in National Health Service (NHS) hospitals and submitted to NELA. This process is described in detail in the NELA annual reports [11]. Individual patient consent is not required as NELA is approved under Section 251 of the NHS Act 2006 by the Confidential Advisory Group. This analysis was performed as part of the NELA project’s remit to understand and improve the care of patients undergoing emergency laparotomy and is exempt from UK National Research Ethics Committee approval as it involves data collected for audit purposes. These data were linked with the Office of National Statistics (ONS) death register for all-cause 30-day mortality.

Patients aged ≥ 18 who had an emergency laparotomy in England and Wales and entered into the NELA database December 2013–December 2020 were eligible for inclusion. Patients undergoing emergency laparotomy for trauma, vascular emergencies, appendicitis and gynaecological complications are excluded from entry into the NELA dataset [18].

The study was reported according to the STROBE guideline for observational studies [19].

#### Outcome variables

Patients in the early-postoperative mortality cohort were compared with all others for age, sex, C-reactive protein (CRP) level, arterial lactate level and frailty. Risk prediction variables American Society of Anesthesiologists (ASA) grade and NELA scores on admission were also examined. The presence of sepsis and markers of septic shock including hypotension and reduced Glasgow Coma Scale (GCS) were recorded. NELA defines sepsis as a National Early Warning Score (NEWS2) of greater than or equal to 5 or greater than or equal to 3 in a single variable [11]. Indications for surgery and surgical findings were compared for both groups.

### Statistical analysis

Descriptive data are presented as median [IQR] or proportion (per cent) as appropriate. Ordinal survey data were analysed using the Kruskal–Wallis test with the Dunn post hoc test to analyse between-group differences. Nominal data were analysed using Fisher’s exact test. *p* values < 0.05 were regarded as statistically significant.

For baseline characteristics of groups, standardised differences of > 0.1 or *p* < 0.05 (using Fisher’s exact or Mann Whitney *U* test as appropriate) were used to suggest baseline imbalance. To assess predictors of futility, a multilevel logistic regression model was conducted incorporating patients nested within hospitals. Clinically important predictors that could be ascertained from initial clinical assessments, were defined a priori and were forced into the multivariate model, with non-significant predictors removed. Predictors included sex, age, ASA grade, high-risk pathology as an indication for surgery (ischaemia or perforation), presence of hypotension, GCS, urgency of surgery, cardiac symptoms and respiratory symptoms, frailty, blood CRP and lactate. Due to non-linearity with continuous predictor variables and logit of the outcome, these were categorised into groups for analysis. All analyses were conducted using Stata^®^ Version 16.1 (StataCorp, www.stata.com).

## Results

### Stage 1: online survey

There were 451 responses from different specialties: surgeons 59%; anaesthetists 29%; geriatricians 7%; intensivists 3%. Two per cent of responses were from junior doctors and advanced nurse practitioners with no declared speciality or had specialty information missing. Two-thirds of the respondents were consultants.

**Fig. 1:**
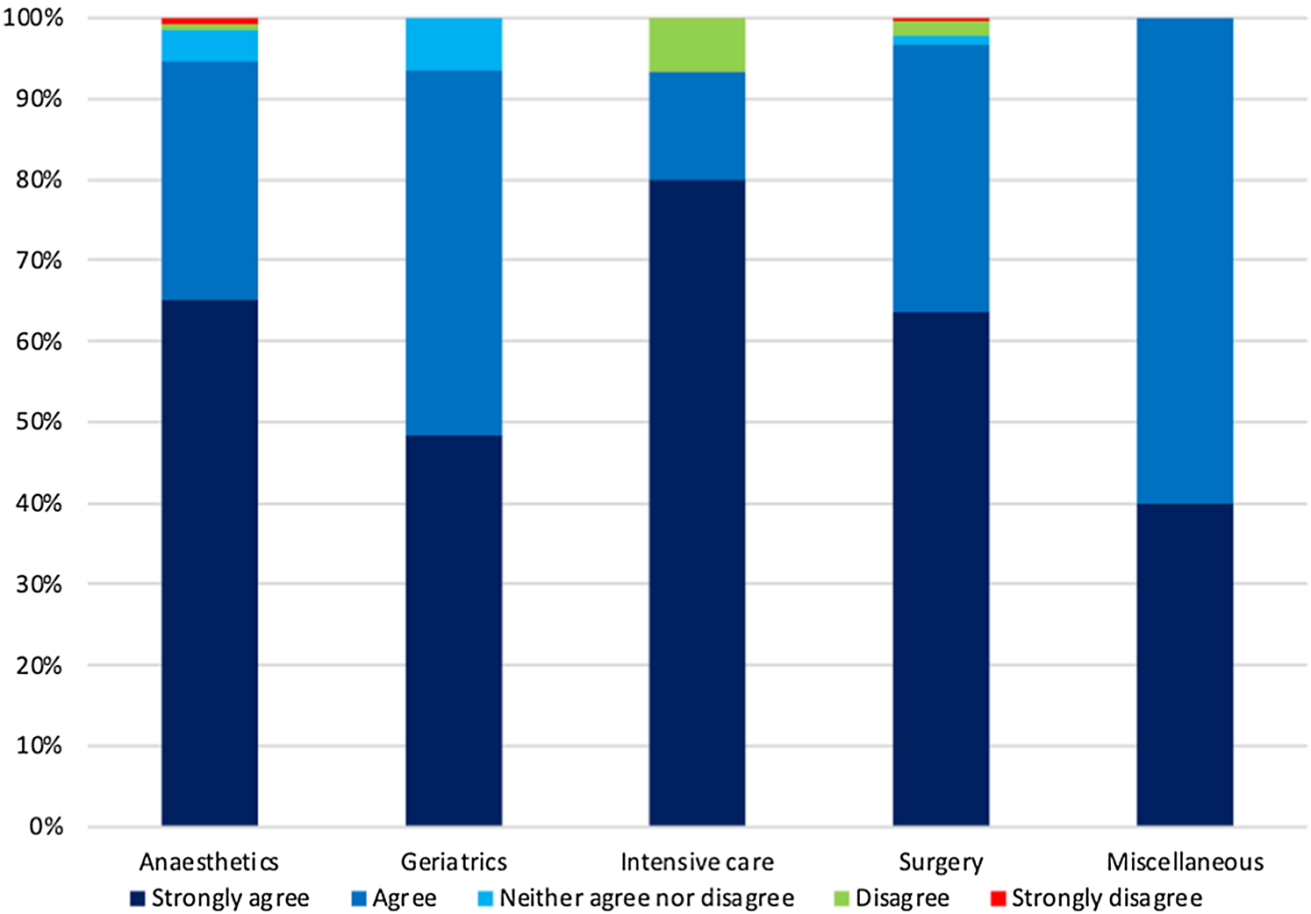
Responses to the question can an emergency laparotomy be futile, by speciality

Almost all respondents agreed that in certain circumstances an emergency laparotomy could be futile: 93% agreed/strongly agreed; 2.4% disagreed (Fig. 1). There was no significant difference in viewpoint between respondents from anaesthetics, surgery or intensive care (*p* = 0.15, 0.44 and 0.13, respectively); however, geriatricians differed significantly from the other three specialties. When asked if an emergency laparotomy could be futile in some circumstances, geriatricians had a median Likert score one point lower than the other groups (*p* = 0.05, 0.05, 0.03 for surgeons, anaesthetists and intensivists, respectively). For patients with poor predicted survival time, the majority of respondents agreed that an emergency laparotomy may be appropriate for palliation of symptoms, with 78% either agreeing/strongly agreeing and only 14% disagreeing.

Respondents were asked to rank which factors were most influential to their decision that an emergency laparotomy may be futile (Table 1). Patient-related factors (encompassing factors such as co-morbidity and frailty) and postoperative survival time were ranked the most important factor by 51% and 36% of respondents, respectively. Surgical pathology was consistently the least important factor (49%), with no difference of opinion by speciality.

**Table 1:**
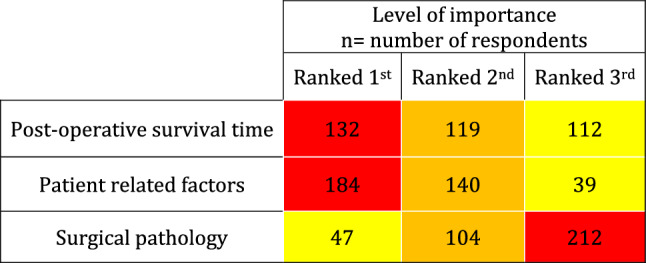
Heatmap of factors that contribute to surgical futility ranked most to least important

Where navy blue is strongly agree, medium blue is agree, light blue is neither agree nor disagree, green is disagree and red is strongly agree

For futile surgery definition, 5% of respondents reported that there was no timeframe that could define futile surgery. For those 95% that defined futile surgery, 9% responded that intraoperative death was their definition of futility, 17% thought a survival time of < 24 h, 51% stated within 72 h and the remaining 18% stated a survival of less than 6 weeks constituted futility. There was no significant difference by speciality.

### Stage 1: scoping review

Three papers examined futility after emergency laparotomy, all of which used quantitative futility as their main outcome. For 2 studies, this was defined as mortality within 72 h of procedure and for 1 within 48 h [12, 14, 15].

### Stage 1: definition of quantitative futility

A consensus definition for a timeframe for quantitative futility was not reached (70%); therefore, a majority decision was considered acceptable. Combining the survey results and the scoping review allowed the steering group to define a timeframe for surgical futility for the purposes of this study as surgery where the patient died within 3 days of the procedure. A timeframe of 3 days was selected as a substitute for 72 h as the NELA dataset records date of death but not time of death.

There was agreement amongst all members of the steering group that < 72 h survival would constitute quantitative futility. Some of the reasons provided from the steering group members for this definition included: the first 72 h after emergency laparotomy are notable as a significant peak of mortality occurs in this window, accounting for approximately 40% of all deaths in the 30 days post-procedure. This timeframe allows for planned reoperation in patients where damage control strategies were utilised and allows a reasonable window for resuscitation in the patient that presented in extremis. Operative complications are a rare cause of early mortality and the majority of complications occur between day three and five [20]; therefore, deaths before day 3are more easily attributable to the underlying pathological process.

### Stage 2: futility in the NELA data

**Fig. 2:**
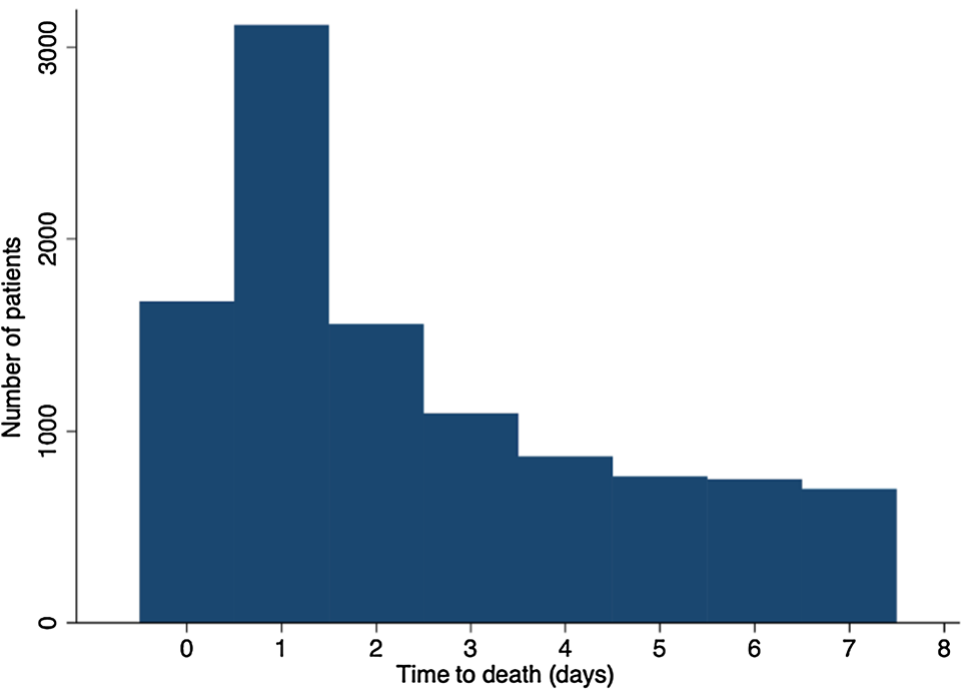
Time to death in the first week post-emergency laparotomy

Of 180,987 patients registered with the NELA database with available mortality data, 7442 (4%) died within 3 days of emergency laparotomy. The modal time of death was 1 day following laparotomy (Fig. 2). The 30- and 90-day mortality of this group of patients was 10.2% and 14.1%, respectively.

#### Characteristics of the early-postoperative mortality cohort

The baseline characteristics of patients in the early-postoperative mortality group compared with the patients who survived the early-postoperative period are displayed in Table 2. Only 3% of these patients were classified as low risk of mortality (NELA score < 5%).

**Table 2:** Baseline characteristics of the two cohorts

	Early postoperative death (*n* = 7442)	Remaining cohort (*n* = 173,545)	Standardised difference (*p* value)
Age (years), median (range)	74 (65–81)	67 (53–77)	0.54 (< 0.001)
Female, *n* (%)	3865/7422 (52)	89,544/173,545 (52)	0.01 (0.57)
From residential or nursing care, *n* (%)	72/2808 (3)	1062/70,692 (1.5)	0.08 (< 0.001)
Rockwood clinical frailty score, *n* (%)			
1–3	529/1743 (30)	31,266/51,221 (61)	0.57 (< 0.001)
4	313/1743 (18)	7220/51,221 (14)	
5	273/1743 (16)	5603/51,221 (11)	
6	476/1743 (27)	5625/51,221 (11)	
7	104/1743 (6)	1155/51,221 (2)	
8	25/1743 (1.4)	207/51,221 (0.4)	
9	23/1743 (1.3)	145/51,221 (0.3)	
ASA grade, median (range)	4 (3–4)	3 (2–3)	1.4 (< 0.001)
P-POSSUM mortality score, median (range)	49.3 (21.2–77.8)	6.1 (2.5–17.7)	1.33 (< 0.001)
NELA mortality score, median (range)	32.4 (17.4–50.3)	3.8 (1.1–10.9)	1.5 (< 0.001)
Lactate (mg/dL), median (range)	4.2 (2.1–7.3)	1.5 (1–2.3)	0.96 (< 0.001)
C-reactive protein (mg/L), median (range)	134 (40–272)	54 (12–171)	0.43 (< 0.001)
Hypotension (systolic < 90 mm/hg), *n* (%)	1590/7362 (22)	5938/172,059 (3)	0.57 (< 0.001)
Severe tachycardia (heart rate > 120 bpm) *n *(%)	1434/7368 (19)	10,099/172,152 (6)	0.42 (< 0.001)
Respiratory signs, *n* (%)			
No shortness of breath	3402/7393 (46)	126,977/172,857 (73)	0.62 (< 0.001)
Shortness of breath on exertion	1896/7393 (26)	28,279/172,857 (16)	
Limiting shortness of breath	1279/7393 (17)	13,105/172,857 (8)	
Shortness of breath at rest	816,7393 (11)	4496/172,857 (3)	
Cardiac signs, *n* (%)			
No cardiac failure	3751/7390 (51)	127,816/172,833 (74)	0.53 (< 0.001)
Diuretic/digoxin/antihypertensive	2371/7390 (32)	35,744/172,833 (21)	
Peripheral oedema	936/7390 (13)	7646/172,833 (4)	
Raised JVP/cardiomegaly	332/7390 (4)	1627/172,833 (1)	

*ASA* American Society of Anaesthesiologists Physical Status Classification System, *P-POSSUM* Portsmouth Physiological and Operative Severity Score for the Enumeration of Mortality, *NEA* National Emergency Laparotomy Audit 30-day mortality risk score, *JVP* jugular venous pressure

Patients in the early-mortality group were older than those who survived the initial postoperative period, with only 7% (491/7442) under the age of 50. Of those, nearly a quarter of these patients had a clinical frailty score of ≥ 5 but most were living in their own home. Eight per cent (13/159) of this younger cohort of patients had a diagnosed learning disability, whereas this figure was 3% in patients under 50 that survived the early-postoperative period (*p* < 0.001).

The three most common indications for surgery in patients with early mortality were peritonitis (36%), followed by perforation (35%) and bowel ischaemia. Bowel ischaemia was an indication in 30% (*n* = 2214) of these patients. In the group that survived the early-postoperative period the most common indications for surgery was small bowel obstruction (37%), followed by perforation (24%), with intestinal ischaemia an indication in just 7% (*n* = 12 145) of cases. Patients who did not survive the initial postoperative period were significantly more likely to have presented with sepsis on admission (56% vs. 30%, *p* < 0.001).

The majority of patients were admitted as an emergency (92%). However, only 72% were admitted under surgery as their receiving speciality compared to 83% in the cohort that survived the initial postoperative period (*p* < 0.001). Patients who had early mortality were less likely to have been seen by surgical consultant preoperatively than their counterparts (93% vs. 96%, *p* < 0.001), but conversely were more likely to have been seen by a consultant anaesthetist preoperatively (72% vs. 60%, *p* < 0.001). These patients were also significantly more likely to have an in-person critical care consultant review than patients who survived the early-postoperative period (41% vs. 14%, *p* < 0.001). Eighty-five per cent of patients who died within 3 days following their procedure were admitted to critical care following their laparotomy (6350/7442). Of the patients that were not admitted to critical care postoperatively, all were classified as high risk [median NELA score 27.7 (IQR 14–45.8)]; however, two-thirds (64%) were recognised as dying during or after their operation and, therefore, were treated palliatively.

Nine per cent (673/7442) of patients in the early-mortality cohort were found to have pathology not amenable to surgery compared to just 0.3% (541/173 545) in the cohort that survived past the first 3 days.

#### Predictors of futile surgery

Table 3 shows the results from the multilevel logistic regression models. Significant predictors of early-postoperative mortality were female sex, increasing age, higher ASA grade, perioperative hypotension, reduced GCS, urgency of surgery and signs of cardiac or respiratory failure and frailty, increasing blood lactate and CRP levels.

**Table 3:** Multilevel, multivariate logistic regression model (*n* = 178,442)

Predictor variable	Odds ratio (95% confidence interval)
Female sex	1.12 (1.06–1.18)
ASA grade	
2	2.04 (1.52–2.73)
3	4.56 (3.42–6.07)
4	13.26 (9.95–17.65)
5	33.51 (24.89–45.12)
Surgical indication—ischaemia	2.67 (2.50 to 2.85)
Surgical indication—perforation	1.55 (1.47 to 1.65)
Age group (age < 50 years as reference)	
50–60 years	1.39 (1.22–1.58)
60–80 years	2.42 (2.17–2.7)
80 + years	3.35 (2.98–3.76)
Hypotension (SBP < 90 mmHg)	2.22 (2.06–2.39)
Glasgow coma scale (GCS 15 as reference)	
13–14	1.68 (1.56–1.82)
8–12	2.25 (1.94–2.59)
3–7	2.82 (2.54–3.15)
Urgency (urgency 18–24 h as reference)	
6–18 h	1.23 (1.08–1.4)
2–6 h	1.88 (1.67–2.12)
> 2 h emergency	1.7 (1.32–2.19)
< 2 h	3.44 (3.03–3.9)
Cardiac signs (no cardiac failure as reference)	
Diuretic/digoxin/hypertension	1.13 (1.06–1.2)
Peripheral oedema	1.34 (1.23–1.47)
Raised JVP/cardiomegaly	1.35 (1.16–1.56)
Respiratory signs (no shortness of breath as reference)	
Shortness of breath on exertion	1.24 (1.16–1.32)
Limiting shortness of breath	1.28 (1.19–1.39)
Shortness of breath at rest	1.39 (1.25–1.53)

*ASA* American Society of Anaesthesiologists Physical Status Classification System, *SBP* systolic blood pressure, *GCS* Glasgow Coma Scale, *JVP* jugular venous pressure

Surgery for intestinal ischaemia was significantly predictive for early-postoperative death (OR 2.67; 95% CI 2.50–2.85) as was intestinal perforation (OR 1.55; 95% CI 1.47–1.65). When frailty was added to the model (*n* = 52,766), it was also a significant multivariate predictor of futility (OR 1.38; 95% CI 1.22–1.55). The further addition of CRP and lactate levels (*n* = 15,918) demonstrated they were also significant multivariate predictors. A CRP of > 100 (OR 2.18; 95% CI 1.15–4.13) and > 200 (OR 2.54; 95% CI 1.36–4.76) approximately doubled the odds of futility compared to a normal CRP level. A lactate of 4–6 (OR 5.27; 95% CI 3.93–7.07) and > 6 (OR 9.51; 95% CI 7.17–12.63) increased the odds of futility by approximately 5 and 10 times, respectively, compared to a normal lactate level.

## Discussion

This work has resulted in a quantitative definition of emergency surgical futility as early-postoperative death within 72 h. Applying this definition to the world’s largest emergency surgery database has reassuringly found only 4% of surgeries result in a quantitatively futile outcome strongly suggesting that appropriate decision-making is part of current emergency surgery practice in the UK.

Combining results of a survey and a scoping review have allowed increased understanding into what perioperative decision-makers think about futility. Although there was a majority agreement from the survey supported by the small number of publications [12, 14, 15], the differing responses from different specialties highlight the varying experiences and values that a multidisciplinary team can provide to these high-risk patients. Where a decision to operate or not may not be clear to a single surgeon, a multidisciplinary council assessment including anaesthetics, critical care and, if appropriate, geriatricians should be routinely considered.

Many of the influencing factors significantly associated with early post-laparotomy mortality are already well known: age, higher blood lactate and CRP, frailty, signs of end organ dysfunction, poor cardiorespiratory reserve and surgery for ischaemia or perforation [12, 16, 21]. Indeed, several are included in the current 30-day mortality risk scoring systems [3–5, 22]. However, none of the risk scoring systems includes frailty and none is tailored to the specific surgical pathology of the patient, only to predicted degree and nature of contamination which may not be immediately obvious from initial CT [3, 4]. Notably, while surgical pathology was a significant predictor of early-postoperative mortality, it was ranked the least important factor to clinicians making the determination that a surgery would be futile when surveyed, suggesting the major role pathology plays in survival is underestimated by clinicians. This is significant as it recognised that the NELA risk prediction score underestimates mortality risk for patients with ischaemia and bleeding [4].

Surgery that resulted in early-postoperative death was most commonly performed on the older patient living with frailty, the patient with poor physiological reserve and the patient presenting in extremis. Whilst it is not surprising that these factors were associated with poorer outcomes, the number of patients operated on in these circumstances is significant; 49% of patients had some level of preoperative cardiac failure, and 28% had limiting exertional breathlessness or breathlessness at rest. Despite the evidence that a patient with a Rockwood Clinical Frailty Score of > 6 is ten times more likely to die in the first 30 days after surgery and seven times more likely to need care on discharge, nearly a third of the patients in the early-mortality cohort were living with this level of frailty or greater [13, 21]. Younger patients in the early-postoperative mortality group were significantly more likely to be frail or have a learning disability than the younger patients that survived the early-postoperative phase. This likely reflects the association these factors have with other health conditions and reduced physiological reserve. However, it is worth noting that this was not absolute and patients living with these factors also survived the early-postoperative period, although their longer term outcome is unknown. Future work to capture the shared decision-making process would be valuable to understand whether the “do everything” attitude and reluctance to suggest palliative care that has been seen in qualitative studies is at play in these decisions [23–26]. The factors associated with early-postoperative mortality were largely predictable, quantifiable and were available to the multidisciplinary decision-making team prior to making the decision to perform an emergency laparotomy. A conscious decision was made by the authors not to create an additional risk score so as not to create a tool used to deny surgery to patients without shared decision-making. Risk scores have flaws in that they are unable to predict individual patient outcomes, and the authors felt there was greater value in defining high-risk markers of early mortality in a quantitative way so that they can be used qualitatively as part of shared decision-making.

A question that remains is whether “failure to rescue” and lack of pre- and intraoperative resuscitation plays a role in early-postoperative mortality. In this study presenting with sepsis worsened outcomes significantly, yet less than a third of these patients who had early mortality received antibiotics in the recommended first hour of presentation [27]. This represents an easily targeted area, that has been proven to reduce mortality [27]. There is also evidence that a > 20% fall from baseline of systolic blood pressure is associated with worse outcomes after emergency laparotomy including kidney injury, cardiac complications and mortality [28, 29]. Yet, a significant proportion of the patients who had early mortality were hypotensive preoperatively. It is not possible to comment on whether this was reversible for each individual patient. However, considering only 68% of patients requiring immediate surgery reach theatre in the appropriate timeframe [11], there may be opportunities in the preoperative period for concurrent resuscitation and surgical workup in order to improve timeliness to theatre and postoperative outcomes.

The Royal College of Surgeons document ‘The high-risk surgical patient’ mandated that patients with a predicted mortality of > 10% should be admitted to critical care postoperatively [30]. Only 85% of patients in the early-mortality group were admitted to critical care for postoperative ongoing resuscitation. While the management strategy for nearly two-thirds of the patients that did not get admitted to critical care was changed to palliative care following their surgery, almost all of the remainder of this group of patients were deemed to be high-risk preoperatively and, therefore, should have been routinely received critical care postoperatively [11]. This highlights the need to keep deaths following emergency laparotomy under constant review. To that end, the authors suggest that all mortality following emergency laparotomy be routinely reviewed at morbidity and mortality meetings to review that key processes and standards are being achieved for these patients.

A limitation of this work is the lack of granularity of the data; as a national collaborative project, it is imperative to minimise the data collection burden. As a result, NELA does not collect data on individual patient comorbidities, details of the decision-making process or cause of death which would be a useful addition to the discussion. In addition, as the questions in the dataset evolve, factors which we now know to be crucial to decision-making and outcomes such as frailty have been added over time and, therefore, are missing from the historical data. There is also a piece of the puzzle missing: patients who do not have emergency laparotomy and instead have palliative management. Even less is known about these patients than patients in the early-postoperative mortality cohort. However, a multicentre cohort study is currently underway looking at their decision-making process and outcomes [31]. Although we examined quantitative futility in this study, it is also important to recognise that to patients there is a spectrum of good and bad outcomes after surgery, not just the dichotomous outcome of survival vs. death. The patient perception of futile surgery was not included in this paper, however, days alive at home, patient-reported outcomes (PROMs) and patient-reported experience measures (PREMS) are increasingly being used as more patient-centred outcome measures following surgery [32–34]. Further patient and public involvement work around the shared decision-making process, as well as the experience of families and carers of patients who did not survive emergency laparotomy, will be vital going forward.

## Conclusions

This study has shown early-postoperative mortality is associated with quantifiable and predictable factors in addition to the known risk factors which make up NELA and NSQIP risk scoring systems. Surgical pathology and frailty are important predictors of poor early-postoperative outcomes, as are extremes of physiological derangement such as perioperative hypotension, lactataemia and preoperative cardiorespiratory dysfunction. This information should be incorporated into shared decision-making conversations with emergency laparotomy patients and their families. Further work to define long-term outcomes following emergency laparotomy examine the patient group that do not have an operation and patient and public involvement work is necessary to understand the nuance of decision-making in these extreme-risk patients.

### Electronic supplementary material

Below is the link to the electronic supplementary material.

Supplementary file1 (DOCX 21 kb)

Supplementary file2 (DOC 80 kb)

Supplementary file3 (DOCX 15 kb)

## Data Availability

Data sharing requests will be considered by the study group upon written request to the corresponding author.

## Appendix

Survey: Defining clinicians’ attitudes and perception of surgical futility in the emergency laparotomy patient.

1. What grade are you?*

(Pick one) *mandatory question

- Foundation doctor
- Advanced nurse or care practitioner
- Surgical trainee
- Anaesthetic trainee
- Health care of older people trainee
- Intensive care trainee
- SAS doctor (healthcare of older people)
- SAS doctor (surgical)
- SAS doctor (anaesthetic)
- SAS doctor (Intensive care)
- Consultant surgeon
- Consultant anaesthetist
- Consultant in intensive care medicine
- Consultant in healthcare of older people

2. To what extent do you agree with the statement “In some circumstance an emergency laparotomy can be futile”?*

(Pick one) *mandatory question

- Strongly agree
- Agree
- Neutral
- Disagree
- Strongly disagree

3. To what extent do you agree with the statement “An emergency laparotomy may be appropriate in a patient with predicted poor survival time for palliation of symptoms”?*

(Pick one) *mandatory question

- Strongly agree
- Agree
- Neutral
- Disagree
- Strongly disagree

4. What factors are most influential to your decision that an emergency laparotomy may be futile?*

(Rank the most important factor 1 and least important factor 3)

*mandatory question

- Predicted post-operative survival time
- Patient factors
- Surgical pathology

5. What post-operative survival time would you consider futile?*

(Pick one) *mandatory question

- Patient death on table
- Death in < 24 h
- Death in < 72 h
- Death in < 6 weeks
- Not applicable

6. What patient factors do you consider most important when making a decision about surgical futility?*

(Rank the most important factor 1 and least important factor 4)

*mandatory question

- Patient age
- Patient frailty
- Patient co-morbidity
- Patient exercise tolerance

7. What surgical pathology would you consider surgically futile?*

(Pick one) *mandatory question

- Incurable malignancy
- Significant GI ischaemia
- Not applicable
- Other free text answer
