# Supplementary material for: Quantitative futility in emergency laparotomy: an exploration of early-postoperative death in the National Emergency Laparotomy Audit
Source: Tech Coloproctol. 2023 Jan 7;27(9):729–38. doi: 10.1007/s10151-022-02747-1 (PMC10404199; doi:10.1007/s10151-022-02747-1)
Supplement: Supplementary file 3 — Supplementary file3 (DOCX 15 kb) [file 10151_2022_2747_MOESM3_ESM.docx]

Defining clinicians’ attitudes and perception of surgical futility in the emergency laparotomy patient

1. What grade are you?*

(Pick one) *mandatory question

Foundation doctor

Advanced nurse or care practitioner

Surgical trainee

Anaesthetic trainee

Health care of older people trainee

Intensive care trainee

SAS doctor (healthcare of older people)

SAS doctor (surgical)

SAS doctor (anaesthetic)

SAS doctor (Intensive care)

Consultant surgeon

Consultant anaesthetist

Consultant in intensive care medicine

Consultant in healthcare of older people

1. To what extent do you agree with the statement “In some circumstance an emergency laparotomy can be futile”?*

(Pick one) *mandatory question

Strongly agree

Agree

Neutral

Disagree

Strongly disagree

1. To what extent do you agree with the statement “An emergency laparotomy may be appropriate in a patient with predicted poor survival time for palliation of symptoms”?*

(Pick one) *mandatory question

Strongly agree

Agree

Neutral

Disagree

Strongly disagree

1. What factors are most influential to your decision that an emergency laparotomy may be futile?*

(Rank the most important factor 1 and least important factor 3) *mandatory question

Predicted post-operative survival time

Patient factors

Surgical pathology

1. What post-operative survival time would you consider futile?*

(Pick one) *mandatory question

Patient death on table

Death in <24 hours

Death in <72 hours

Death in <6 weeks

Not applicable

1. What patient factors do you consider most important when making a decision about surgical futility?*

(Rank the most important factor 1 and least important factor 4) *mandatory question

Patient age

Patient frailty

Patient co-morbidity

Patient exercise tolerance

1. What surgical pathology would you consider surgically futile?*

(Pick one) *mandatory question

Incurable malignancy

Significant GI ischaemia

Not applicable

Other free text answer
